# Refining post-neoadjuvant risk stratification in ESCC with lymph node regression grade

**DOI:** 10.3389/fonc.2026.1723139

**Published:** 2026-05-13

**Authors:** Wenxin Liang, Maohui Chen, Deyong Kang, Hongjin Wang, Rui Tong, Chun Chen, Wei Zheng

**Affiliations:** 1 Department of Thoracic Surgery, Fujian Medical University Union Hospital, Fuzhou, Fujian, China; 2 Key Laboratory of Cardio-Thoracic Surgery (Fujian Medical University), Fujian Province University, Fuzhou, Fujian, China; 3 National Key Clinical Specialty of Thoracic Surgery, Fuzhou, China; 4 Department of pathology, Fujian Medical University Union Hospital, Fuzhou, Fujian, China; 5 Department of Cardiovascular Surgery, Longyan First Affiliated Hospital of Fujian Medical University, Longyan, China

**Keywords:** esophageal squamous cell carcinoma, neoadjuvant chemotherapy, risk stratification, tumor prognosis, tumor regression grade

## Abstract

**Background:**

Neoadjuvant chemotherapy (NAC) followed by surgery is standard care for locally advanced esophageal squamous cell carcinoma (ESCC), yet reliable post-NAC prognostic indicators remain limited. We evaluated the prognostic value of lymph node regression grade (LRG) in ESCC after NAC.

**Methods:**

We retrospectively reviewed 173 patients with ESCC who received NAC followed by resection at Fujian Medical University Union Hospital (2013–2018). Based on LRG, patients were categorized as lymph-node response (LN-R), lymph-node non-response (LN-NR), or lymph-node negative (LN-Neg). Kaplan–Meier analyses compared overall survival (OS) and disease-free survival (DFS) among groups. Variables with P<0.05 in univariable Cox models, together with clinically relevant covariates, entered multivariable Cox regression. Nomograms for OS and DFS were developed from multivariable results; calibration curves were used to assess agreement between predicted and observed outcomes.

**Results:**

Five-year OS differed significantly across groups: 69.2% (LN-R), 57.4% (LN-Neg), and 24.6% (LN-NR) (P<0.001). DFS also varied significantly among the three groups (P<0.001). On multivariable analysis, LN-R status was independently associated with lower risks of death (hazard ratio=0.38) and recurrence, supporting LRG as a favorable prognostic factor. Poor differentiation (G2-G3) and advanced ypT stage (3-4) were adverse prognosticators. Nomograms incorporating LRG and pathological factors were constructed and calibrated.

**Conclusions:**

LRG provides independent prognostic information in ESCC after NAC, beyond primary-tumor response. Incorporating LRG into post-NAC risk stratification may refine prognostic assessment and help guide individualized management in the precision-medicine era.

## Introduction

Esophageal cancer is one of the most prevalent malignant tumors worldwide, causing over 500,000 patient deaths every year (1). Esophageal cancer has a high malignancy and strong invasive capability, with lymph node metastasis often occurring at an early stage (2). Due to the lack of early clinical symptoms, esophageal cancer is typically diagnosed at an advanced stage, leading to a poor prognosis (3). Esophageal cancer pathologic subtypes mainly include esophageal squamous cell carcinoma(ESCC) and esophageal adenocarcinoma, each showing significant differences in treatment strategies and prognosis (4).

Preoperative neoadjuvant chemoradiotherapy (NCRT) or neoadjuvant chemotherapy (NAC) followed by esophagectomy is the recommended standard treatment for advanced ESCC patients (5–8). Furthermore, neoadjuvant therapy can reduce cancer staging, eradicate micrometastases, and increase the probability of achieving resectability and curative resection (9). It is worth noting that not all patients respond to NAC, and whether survival benefits can be achieved from NAC treatment is also a consideration. Therefore, determining effective prognostic indicators for patients after NAC treatment is of significant clinical value for making precision diagnostic and therapeutic decisions. It is important to note that the value of some lymph node-related pathological features in NAC treatment has been established. For example, research evaluating prognostic data of metastatic esophageal cancer patients has found that lymph node regression to NAC, compared to the primary tumor response to NAC, is more accurate in predicting long-term survival rates (10).

Studies have also shown that esophageal cancer patients with lymph node metastases may exhibit some resistance to NAC (11). Some previous studies indicated that lymph node regression following NAC is an important prognostic indicator for patients (12, 13). Similarly, studies have indicated that patients who exhibit lymph node regression following neoadjuvant treatment have a reduced mortality rate compared to those with no regression or unclear regression of lymph nodes (14, 15). However, the aforementioned studies have primarily focused on esophageal adenocarcinoma, and the prognostic value of lymph node regression in the diagnosis and treatment of ESCC patients requires further investigation. Additionally, assessing whether combining other clinical pathological parameters can lead to further effective stratification of prognosis also needs to be explored.

In this study, we aim to evaluate the prognosis value of lymph node regression in ESCC patients after NAC by quantifying lymph node regression status using lymph node regression grade (LRG). This study intends to use patient sample data from our center to employ various methods to explore the relationship between LRG and patient prognosis, as well as to determine whether it can complement the prognostic value of primary tumor regression.

## Methods

### Research population

This study selected patients with locally advanced thoracic ESCC who were treated at the Department of Thoracic Surgery, Fujian Medical University Union Hospital between 2013 and 2018. The inclusion criteria were as follows: (i) patients with a pathological diagnosis of ESCC prior to neoadjuvant chemotherapy, (ii) clinical stage cT3–4 or cN-positive, (iii) aged between 18 and 70 years, (iv) ECOG performance status of 0 or 1, (v) Patients received 2–4 cycles of preoperative neoadjuvant treatment, followed by McKeown three-incision esophagectomy with either two-field or three-field lymphadenectomy, achieving R0 resection. Exclusion criteria were as follows: (i) patients with other malignancies, (ii) incomplete clinical data, (iii) Patients who died within 30 days after surgery.

All patients completed a standardized preoperative assessment protocol comprising physical examination, laboratory investigations (complete blood count and serum biochemistry), and contrast-enhanced computed tomography (CT) of the neck, chest, and abdomen, bilateral supraclavicular lymph node ultrasonography, endoscopic ultrasound, and whole-body fluorodeoxyglucose positron emission tomography/CT (FDG PET/CT). Based on National comprehensive cancer network (NCCN) guidelines, NAC was administered to locally advanced ESCC patients, consisting of paclitaxel at 135mg/m² infused intravenously for over 3 hours, and cisplatin or carboplatin at 75mg/m² infused intravenously for 1–2 hours. In this study, we preferentially selected cisplatin and used carboplatin only for patients at higher risk of nephrotoxicity or ototoxicity. Each cycle was repeated every 3 weeks for 2–4 cycles, followed by surgery 4–8 weeks after NAC. All patients received McKeown minimally invasive esophagectomy. The 2-field lymphadenectomy was regularly performed, and 3-field lymphadenectomy was conducted when patients with suspected positive LNs within the cervical area.

### Histopathological examination and pathological evaluation

All patients who underwent surgical resection had their tumor pathological tissues reviewed by senior pathologists, resulting in final histological examination results.

Two senior pathologists, who were blinded to the patients’ clinical outcomes, reassessed the pathological sections independently, primarily focusing on tumor regression grade (TRG) and LRG. The interobserver agreement of the two grading systems was quantitatively analyzed using Cohen’s Kappa statistics. The results confirmed that the Kappa values for both LRG and TRG assessments reached 1.00, indicating perfect consistency between the two observers in grading determination. The primary TRG was graded according to the Becker classification, with TRG0 indicating complete pathological response. Residual tumors <10%,10%-50%,>50% were classified as TRG1, TRG 2, and TRG 3, respectively.

The lymph node response score was modified from a previously published grading system (13): (i) score 0 for complete remission, (ii) score 1indicates <50% viable residual tumor in positive lymph nodes,(iii) score 2 indicates >50% viable residual tumor in positive lymph nodes. Negative lymph nodes were also recorded, indicating no evidence of regression or pre-cancerous changes. Regarding lymph nodes without metastasis but with regressive changes, they were defined as lymph nodes in which no viable tumor cells, residual tumor tissue, or traces of degenerated tumor cells were identified by pathological examination; however, these nodes exhibited morphological changes such as focal fibrosis, lymphoid hyperplasia, or scattered inflammatory cell infiltration following NAC. Such regressive changes indicate a therapeutic effect but, in the absence of viable tumor cells, meet the pathological criteria for a negative nodal status. From a study design perspective, our LRG system specifically targets the response of metastatic lymph nodes. Therefore, lymph nodes without pretreatment metastasis, regardless of post-treatment regressive changes, were classified into the LN-Neg group to avoid confounding the regression grading of truly positive nodes. Based on the core design principle of this study, where LRG focuses on the therapeutic response of metastatic lymph nodes, Such lymph nodes were ultimately classified as negative lymph nodes. ESCC patients were stratified into three groups based on LRG assessment and nodal status: (i) Lymph node negative (LN-Neg) group: patients without pathological lymph node metastasis, (ii) Lymph node response (LN-R) group: patients with metastatic lymph nodes showed LRG scores of 0 or 1, (iii) Lymph node non-response (LN-NR) group: patients with metastatic lymph nodes showed LRG score of 2.In cases where multiple lymph nodes from the same patient showed different regression results, any lymph node with a regression score of 2 was included in the LN-NR group (16, 17).The simplified classification (LN-R, LN-NR, LN-Neg) was chosen based on two main considerations. First, it strikes a balance between prognostic discrimination, clinical practicality, and pathological reproducibility. Second, grouping LRG 0 and LRG 1 into the LN-R group was due to the limited sample size in our single-center cohort; further splitting these two subgroups would result in insufficient sample size for meaningful statistical analysis. This approach is consistent with previous studies showing comparable prognosis between LRG 0 and LRG 1 patients after neoadjuvant therapy.

### Follow-up

Patients were followed up every 3 months within 2 years postoperatively, semi-annually between 2–5 years, and annually thereafter until death or lost to follow-up. Follow-up assessments included physical examinations, neck/chest/abdominal CT scans, cervical and abdominal ultrasound, laboratory tests, upper gastrointestinal contrast imaging, whole-body FDG PET/CT, bone scans, cranial MRI, and endoscopic examinations based on the patient’s postoperative condition changes. Overall survival (OS) was defined as the time from the date of surgery to death from any cause or last follow-up date, while disease-free survival (DFS) was defined as the time from the date of surgery to disease recurrence or death from any cause.

### Nomogram development and validation

Based on the final multivariable Cox regression models for OS and DFS, two separate nomograms were constructed to provide individualized probability predictions. The variables incorporated into the nomograms were selected based on their statistical significance in the multivariable analysis and clinical relevance.Internal validation of the nomograms was performed using bootstrap resampling with optimism correction to assess and adjust for model overfitting. Specifically, 1000 bootstrap samples were drawn with replacement from the original cohort. In each bootstrap sample, the Cox models were refitted, and their predictive performance was evaluated. The optimism (the difference between the performance in the bootstrap sample and its performance when applied to the original sample) was estimated for each performance measure. The final reported discrimination and calibration metrics were corrected by subtracting this estimated optimism. Discrimination was quantified using the optimism-corrected Harrell’s concordance index. Calibration, which evaluates the agreement between predicted and observed survival outcomes, was assessed using calibration curves generated from the bootstrap-corrected models and by the Hosmer-Lemeshow goodness-of-fit test adapted for censored time-to-event data. Furthermore, to assess the utility of the models in clinical decision-making, decision curve analyses were performed for the three models at the 3-year and 5-year time points, with OS as the endpoint.

### Statistical analysis

In this study, sporadic missing data were handled using mean imputation for continuous variables and mode imputation for categorical variables. Fisher’s and χ2 tests were used for analyzing differences in categorical variables, while t-tests and Mann-Whitney U tests were used for continuous variables. Cox regression models and Kaplan-Meier methodology were employed to evaluate potential associations between clinical characteristics and both OS and DFS. Multivariable Cox analysis was conducted on significantly associated clinical features from single-variable analysis. Calibration curves visualized the consistency of model predictions with actual patient survival outcomes. Good calibration was indicated by a P-value > 0.05 in the Hosmer-Lemeshow goodness-of-fit test. Statistical significance was determined at P < 0.05 for all analyses. All statistical analyses were performed using R version 4.2.1.

## Results

### Baseline characteristics of enrolled patients

According to our inclusion and exclusion criteria, a total of 173 patients met the criteria and were included in the analysis. Male patients accounted for 83.8% of cases, with an overall median age of 57 years. Lymph node dissection yielded a median of 32 nodes (range: 6-94). Among them, 58.96% of patients had lymph node metastasis. Posttherapy pathological staging was determined using AJCC 8th edition criteria, with nodal classifications distributed as follows: ypN0 (49.1%), ypN1 (28.3%), ypN2 (13.9%), ypN3 (8.7%).The distribution of treatment cycles was as follows: 85% (147 patients) completed 3 cycles of preoperative neoadjuvant chemotherapy, 9.8% (17 patients) completed 2 cycles, and 5.2% (9 patients) completed 4 cycles.

### Response of NAC treatment

After evaluation of pathological sections by experienced pathologists, the treatment response of the primary tumor was graded according to the Becker grading system. 26 patients (15.03%) achieved pathological complete response of the primary tumor, classified as TRG0. And 30 patients (17.34%), 63 patients (36.42%), and 54 patients (31.21%) were classified as TRG1, TRG2, and TRG3, respectively (**Table 1**).

**Table 1:** Clinicopathological parameters of included patients.

Parameters	Lymph node response (n=33)	Lymph node non-response (n=69)	Lymph node negative (n=71)	P-value
Age	57.22 ± 5.97	55.01 ± 6.90	57.75 ± 6.22	0.039
BMI (kg/m2)	21.78 ± 2.57	22.02 ± 3.23	22.09 ± 2.82	0.9
Harvested lymph nodes	31.91 ± 12.24	38.62 ± 14.73	31.15 ± 12.79	0.003
Positive lymph nodes	1.42 ± 1.92	4.78 ± 6.23	0	< 0.001
Sex				0.3
male	28 (84.8)	61 (88.4)	56 (78.9)	
female	5 (15.2)	8 (11.6)	15 (21.1)	
Tumor location				0.041
upper	4	12	16	
middle	19	39	44	
lower	10	18	11	
Lymphadenectomy				0.4
2-field	22 (66.7)	45(65.2)	53 (74.6)	
3-field	11 (33.3)	24(34.8)	18 (25.4)	
ypTNM				< 0.001
PCR	3	0	16	
I	4	1	23	
II	4	4	29	
III	20	50	2	
IV	2	14	1	
Becker score				< 0.001
0	6	2	18	
1	10	7	13	
2	13	30	20	
3	4	30	20	

BMI, body mass index.

**Figure 1:**
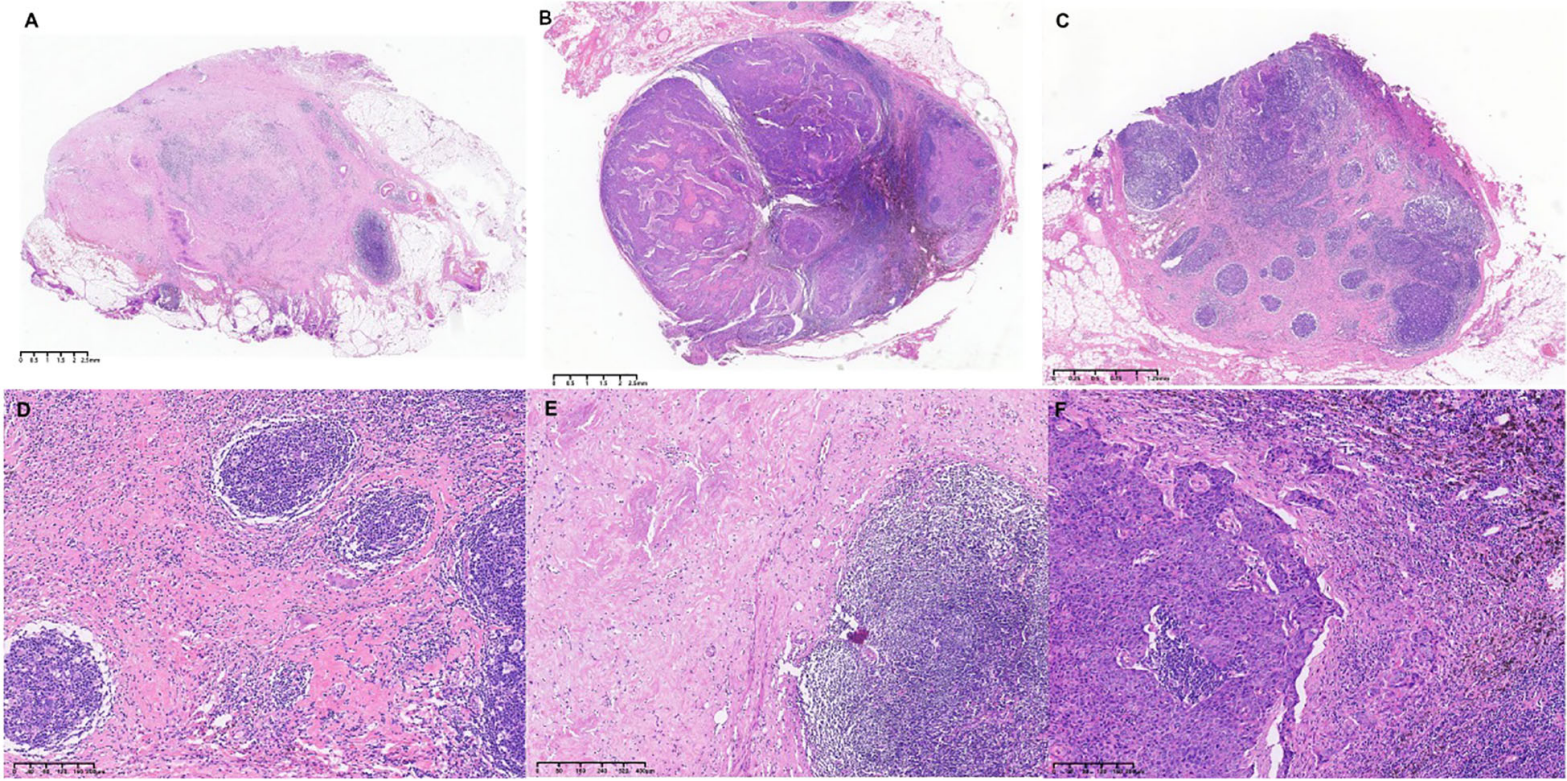
Hematoxylin-eosin staining of lymph node regression score. **(A)** The score is 0, and there are no tumor cells in the lymph nodes after neoadjuvant treatment. **(B)** Score 1, the proportion of fibrous tissue and residual tumor cells in the lymph nodes after neoadjuvant treatment is less than 50%. **(C)** Score 2, the proportion of fibrous tissue and residual tumor cells in the lymph nodes after neoadjuvant treatment is greater than 50%. **(D)** 10×10x magnification for image **(A, E)** 10×10x magnification for image **(B, F)** 10×10x magnification for image C.

Lymph node pathological sections of 173 patients were reviewed, and lymph nodes with responses were scored based on the ratio of fibrous tissue to residual tumor within the lymph node. A total of 5930 lymph nodes were reviewed, with 5501 lymph nodes (92.76%) evaluated as having no tumor metastasis before treatment. 51 lymph nodes (0.86%) were scored as LRG0, indicating no residual tumor within the lymph node after treatment. 96 lymph nodes (1.62%) were scored as LRG1, and 282 lymph nodes (4.76%) were scored as LRG2. Typical pathological images of lymph node regression scoring LRG0–2 are shown in **Figure 1**.

### Relationship between LRG and ESCC patients’ prognosis

**Figure 2:**
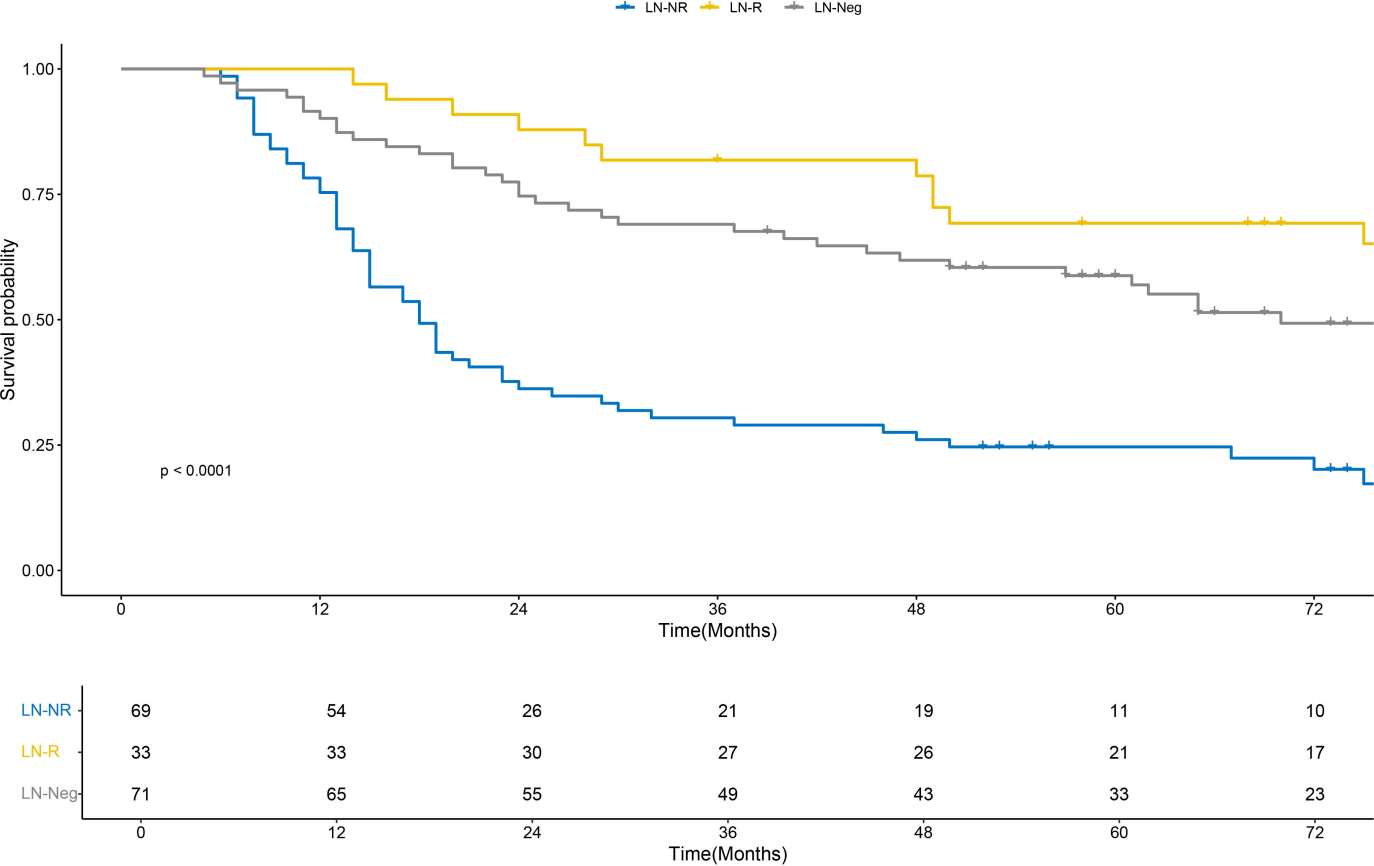
K-M plots show the OS of patients with different lymph node regression statuses. OS, overall survival; K-M, Kaplan-Meier.

**Figure 3:**
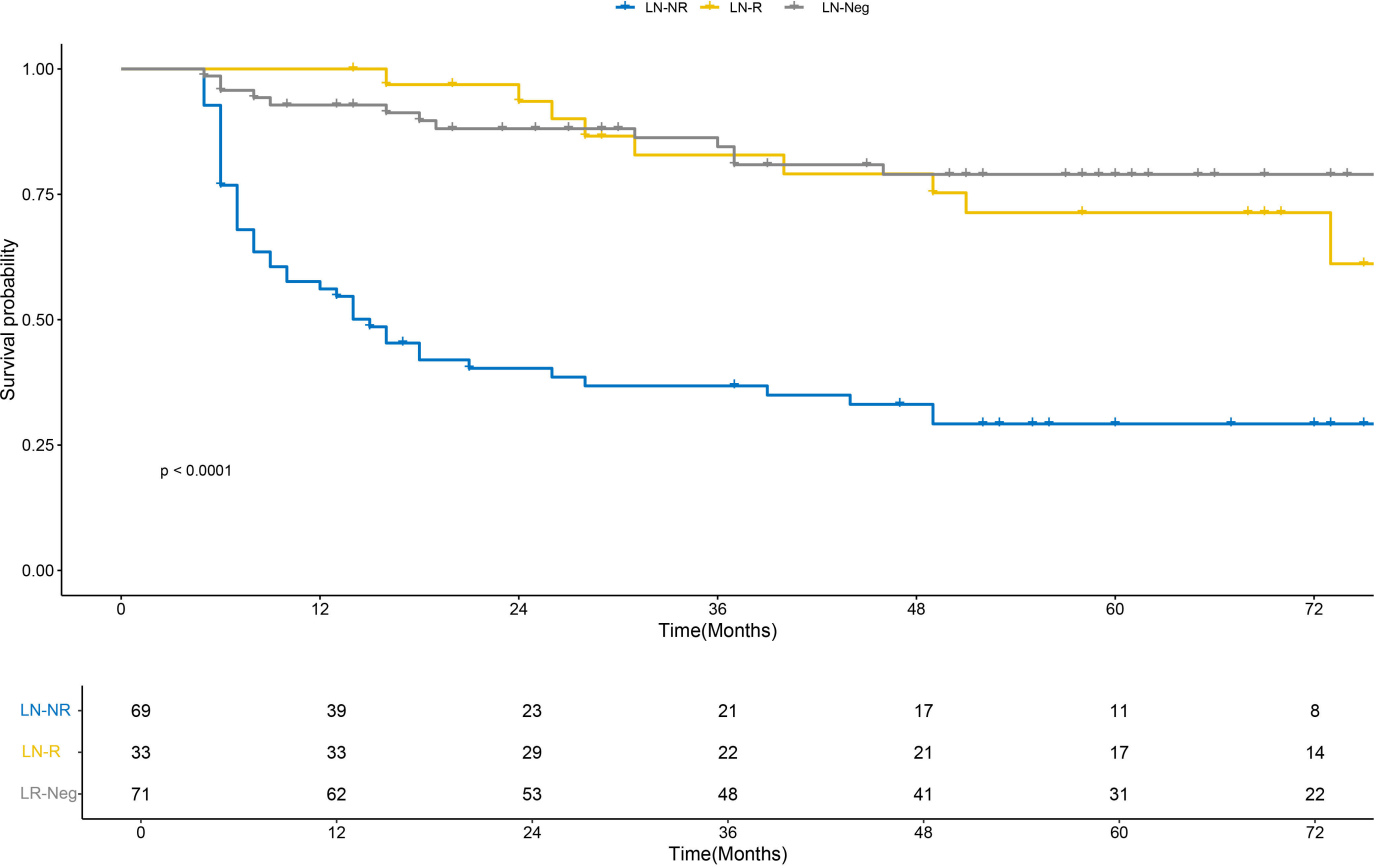
K-M plots show the DFS of patients with different lymph node regression statuses. DFS, disease-free survival; K-M, Kaplan-Meier.

Event statistics for the study cohort are as follows: for OS (total cases = 173), 112 death events were recorded (event rate = 64.7%) with a median follow-up duration of 74 months; for DFS (total cases = 173), 70 events (including recurrence or death) were documented (event rate = 40.5%) with a median follow-up duration of 62 months (**Supplementary Table 8**). Kaplan-Meier curve analysis revealed that the LN-R group and the LN-Neg group exhibited significantly superior OS outcomes compared with the LN-NR group, with statistically significant differences evident in the survival curves across these groups (**Figure 2**). For DFS, while the LN-Neg group retained the favorable prognostic profile, the LN-R group demonstrated a higher survival probability than the LN-NR group (**Figure 3**). These observations suggest that LRG confers a positive influence on DFS. With regard to OS the median survival time was 51.5 months in the LN-R group, 48 months in the LN-Neg group, and 42.5 months in the LN-NR group. For DFS, the median survival time was 46.5 months in the LN-R group, 37 months in the LN-Neg group, and 31 months in the LN-NR group.

Univariate Cox analysis demonstrated that the LN-R group had significantly better prognosis in terms of OS and DFS compared with the LN-NR group (**Tables 2**, **3**). In the univariate analysis, for OS, with the LN-NR group as the reference, the LN-R group showed a reduced risk of event occurrence (HR = 0.248, 95% CI: 0.143 - 0.430, P < 0.001). Similarly, the LN-Neg group also exhibited favorable prognosis (HR = 0.305, 95% CI: 0.200 - 0.467, P < 0.001). These results indicated that both the LN-R group and the LN-Neg group were protective factors for prognosis. Regarding DFS, LRG also showed a significantly relevant prognostic impact. With the LN-NR group as the reference (HR = 1.000), both the LN-R group (HR = 0.249, 95% CI: 0.125-0.497, P<0.001) and the LN-Neg group (HR = 0.179, 95% CI: 0.098 - 0.327, P < 0.001) exhibited a lower risk of DFS event occurrence. Beyond LRG, several other factors were significantly associated with poorer OS, including advanced pathological T stage (ypT3–4), lymph node metastasis (ypN1–3), poor tumor differentiation (Grade 2–3), and suboptimal primary tumor regression (TRG2–3). For DFS, in addition to the factors above, age > 60 years was also significantly correlated with inferior outcomes. For the risk factor analysis of OS and DFS in ypN(+) patients (see **Supplementary Tables 3**–**5**), LRG demonstrated stable and statistically significant prognostic value for both endpoints in this restricted analysis, with consistent direction and magnitude of its risk effect observed in both univariate and all multivariate models.

**Table 2:** Univariate and multivariate cox analysis determine relationships between clinicopathological parameters and patients’ OS.

Variables	Number	Univariate analysis			Multivariate analysis		
		HR	95%CI	P value	HR	95%CI	P value
Age							
<=60	120	1.000			1.000		
>60	53	1.738	1.172-2.578	0.006	1.282	0.818-2.011	0.279
Sex							
Male	145	1.000					
Female	28	0.988	0.602-1.619	0.961			
BMI							
<=23	112	1.000					
>23	61	1.011	0.687-1.489	0.956			
Location							
Upper	32	1.000					
Middle	102	0.858	0.533-1.382	0.528			
Low	39	0.717	0.401-1.279	0.260			
Total number of lymph nodes dissected							
	173	1.002	0.988-1.015	0.802			
ypT stage							
0-2	81	1.000			1.000		
3-4	92	2.186	1.485-3.218	< 0.001	1.770	1.176-2.665	0.006
ypN stage							
0	85	1.000			1.000		
1	49	1.738	1.106-2.731	0.017	1.368	0.694-2.694	0.366
2	24	4.714	2.760-8.054	< 0.001	1.934	0.868-4.306	0.106
3	15	4.125	2.230-7.629	< 0.001	2.560	1.152-5.692	0.021
Tumor differentiation							
1	85	1.000			1.000		
2	45	2.754	1.715-4.421	< 0.001	2.250	1.367-3.702	0.001
3	43	4.178	2.626-6.648	< 0.001	3.018	1.848-4.929	< 0.001
TRG							
0	26	1.000					
1	30	1.660	0.753-3.662	0.209			
2	63	2.487	1.244-4.972	< 0.001			
3	54	4.824	2.414-9.640	< 0.001			
LRG							
LN-NR	69	1.000			1.000		
LN-R	33	0.248	0.143-0.430	< 0.001	0.380	0.211-0.683	0.001
LN-Neg	71	0.305	0.200-0.467	< 0.001	0.523	0.265-1.030	0.061

OS, overall survival; HR, Hazard ratio; CI, Confidence Interval; TRG, tumor regression grade; LRG, Lymph node regression grade; LN-NR, Lymph node non-response; LN-R, Lymph node response; LN-Neg, Lymph node negative.

**Table 3:** Univariate and multivariate cox analysis determine relationships between clinicopathological parameters and patients’ DFS.

Variables	Number	Univariate analysis			Multivariate analysis		
		HR	95%CI	P value	HR	95%CI	P value
Age							
<=60	120	1.000			1.000		
>60	53	1.688	1.033-2.757	0.037	1.288	0.735-2.355	0.377
Sex							
Male	145	1.000					
Female	28	0.585	0.280-1.223	0.154			
BMI							
<=23	112	1.000					
>23	61	0.846	0.513-1.394	0.511			
Location							
Upper	32	1.000					
Middle	102	1.216	0.607-2.437	0.581			
Low	39	1.628	0.767-3.458	0.205			
Total number of lymph nodes dissected							
	173	1.001	0.985-1.018	0.864			
ypT stage							
0-2	81	1.000			1.000		
3-4	92	2.363	1.439-3.881	0.001	1.887	1.123-3.171	0.017
ypN stage							
0	85	1.000			1.000		
1	49	2.137	1.183-3.863	0.012	0.959	0.448-2.050	0.914
2	24	5.423	2.755-10.675	< 0.001	1.257	0.501-3.152	0.626
3	15	5.304	2.551-11.027	< 0.001	1.863	0.769-4.513	0.168
Tumor differentiation							
1	85	1.000			1.000		
2	45	2.034	1.141-3.627	0.016	1.666	0.889-3.120	0.111
3	43	2.976	1.671-5.302	< 0.001	2.211	1.183-4.130	0.013
TRG							
0	26	1.000					
1	30	3.112	1.003-9.652	0.049			
2	63	3.670	1.279-10.526	0.016			
3	54	6.283	2.193-17.999	0.001			
LRG							
LN-NR	69	1.000			1.000		
LN-R	33	0.249	0.125-0.497	< 0.001	0.352	0.169-0.737	0.006
LN-Neg	71	0.179	0.098-0.327	< 0.001	0.217	0.096-0.493	< 0.001

DFS, Disease-free survival; HR, Hazard ratio; CI, Confidence Interval; TRG, tumor regression grade; LRG, Lymph node regression grade; LN-NR, Lymph node non-response; LN-R, Lymph node response; LN-Neg, Lymph node negative.

### LRG as an independent prognostic indicator for patients

Multivariate Cox analysis was conducted to clarify whether LRG was an independent risk factor for patients (**Tables 2**, **3**). For OS, the LN-R group exhibited a significant independent protective effect: with the LN-NR group as the reference, the HR of the LN-R group was 0.380 (95% CI: 0.211-0.683, P = 0.001). This suggests that patients with lymph node reactive characteristics have a significantly reduced risk of death, and LRG can serve as one of the core indicators for OS prognostic stratification. In the OS analysis of this study, ypN2 was significantly associated with OS in univariate analysis, but its effect weakened and lost statistical significance (P = 0.106) after incorporating LRG and other covariates into the multivariate Cox regression model, while LRG remained significant. This result suggests that the predictive role of LRG in OS surpasses that of simple lymph node staging to a certain extent, highlighting its strong independent prognostic value and thus further reflecting the clinical significance and application value of this study. The multivariate analysis of DFS further verified the prognostic value of LRG: the HR of the LN-R group was 0.352 (95% CI: 0.169-0.737, P = 0.006), and the HR of the LN-Neg group was 0.217 (95% CI: 0.096-0.493, P < 0.001), both of which independently reduced the risk of tumor recurrence.

### Constructing nomograms to integrate prognostic clinical feature parameters

**Figure 4:**
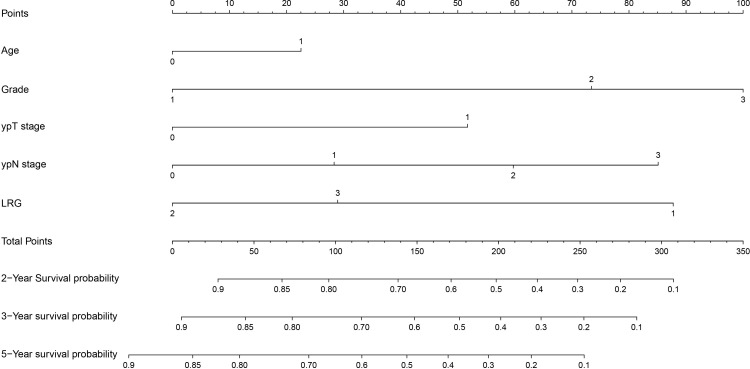
For the analysis of patients’ OS data, we constructed a prognostic model based on the number of Age, Grade, ypT stage, ypN stage, LRG. OS, overall survival; LRG, lymph node regression grade.

**Figure 5:**
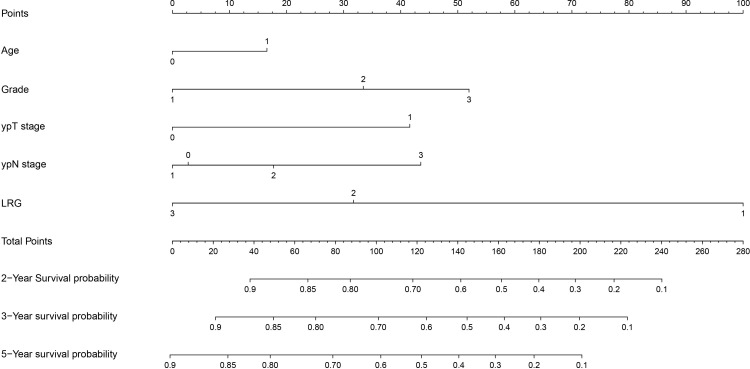
For the analysis of patients’ DFS data, we constructed a prognostic model based on the number of Age, Grade, ypT stage, ypN stage, LRG. DFS, disease-free survival; LRG, lymph node regression grade.

To further integrate multiple clinical features and construct a comprehensive prognostic model for more accurate prediction of outcomes in patients with esophageal squamous cell carcinoma (ESCC), this study developed a dual-endpoint nomogram for overall survival (OS) and disease-free survival (DFS). Initially, all variables with statistical significance in the univariate analysis (**Supplementary Table 1**) were incorporated into the multivariable Cox model. In this full-variable model, both TRG and ypT stage lost statistical significance, suggesting substantial overlap in the prognostic information carried by TRG with other post-neoadjuvant response-related variables. This was further supported by the variable correlation heatmap (**Supplementary Figure 1**), which demonstrated a moderate-to-strong correlation between ypT stage and TRG (r =0.651), confirming the potential collinearity between these two variables. To address this, sensitivity analyses were performed by constructing alternative multivariable models, where one correlated response-related variable was excluded at a time (**Supplementary Table 2**). Results showed that the independent association between LRG and outcomes remained stable across all alternative models, whereas the effect of TRG lacked robustness after adjustment. To avoid collinearity and maintain the parsimony of the nomogram, LRG (rather than TRG) was retained in the final nomogram. This model incorporates key clinical characteristics, including Age, Tumor differentiation, ypT stage, ypN stage, and LRG. It enables personalized prediction of OS (**Figure 4**) and DFS (**Figure 5**) in ESCC patients, facilitating the identification and risk stratification of these patients as well as guiding precise clinical follow-up.

**Figure 6:**
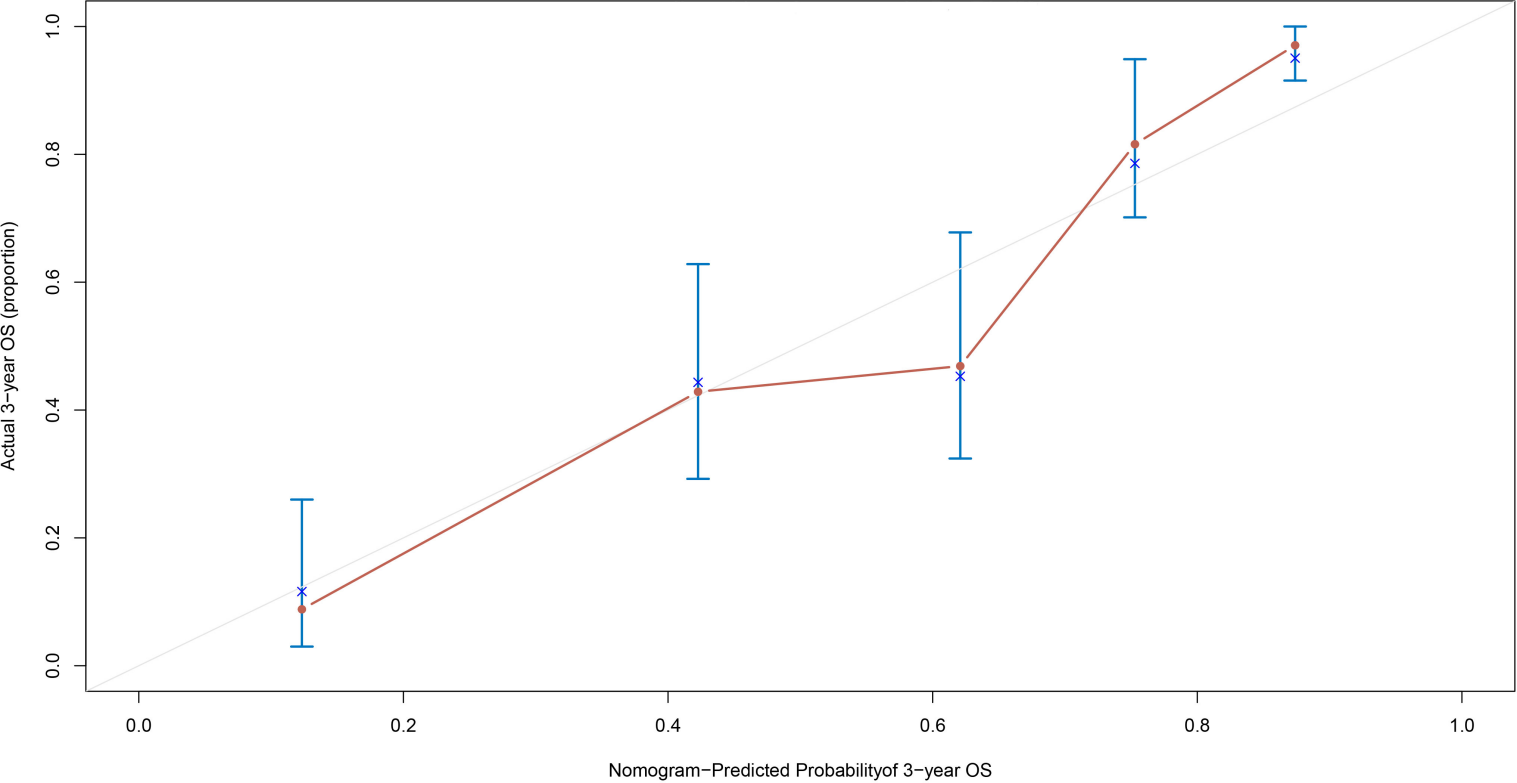
The 3-year OS calibration curve. OS, overall survival.

For the OS nomogram, the overall concordance index (C-index) was 0.770, indicating a moderate ability to discriminate between patients with different survival risks. The 3-year time-specific C-index was further improved to 0.849, which significantly enhanced the accuracy of identifying short-term mortality risk. Additionally, in the 3-year OS calibration curve (**Figure 6**), the red predicted curve showed a high degree of agreement with the ideal reference line, and the error bars (95% CI) confirmed good consistency between the predicted values and actual survival proportions. Decision curve analysis at 3-year (**Supplementary Figure 2**) and 5-year (**Supplementary Figure 3**) further demonstrated favorable clinical utility of the nomogram across a range of risk thresholds for OS prediction.

**Figure 7:**
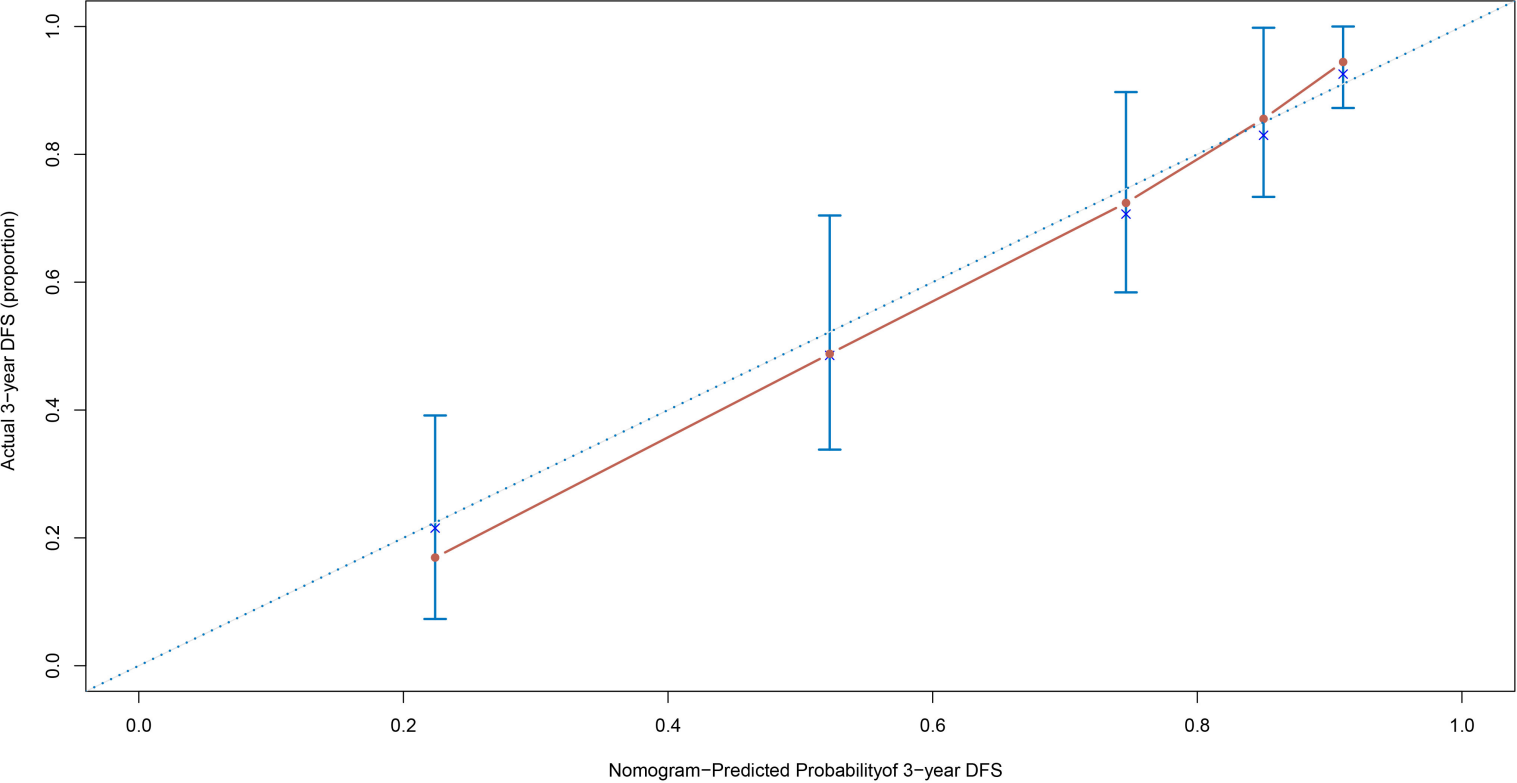
The 3-year DFS calibration curve. DFS, disease-free survival.

For the DFS nomogram, the overall C-index was 0.795, which effectively stratified patients based on DFS prognostic differences. The 3-year time-specific C-index increased to 0.874, resulting in superior accuracy for predicting short-term disease-free survival risk. In the 3-year DFS calibration curve (**Figure 7**), the red predicted curve overlapped with the ideal line, and the error bars (95% CI) reflected high reliability between the predicted values and actual disease-free survival outcomes.

It is noteworthy that LRG played a critical role in both nomograms and was identified as one of the key prognostic factors. Collectively, these two models provide practical tools for individualized risk assessment, follow-up planning, and treatment decision-making in ESCC patients, thereby supporting precise clinical management.

## Discussion

With the development of NAC strategies, determining post-NAC prognostic indicators for ESCC patients holds significant clinical importance. In the present study, we systematically investigated the prognostic value of LRG in patients with ESCC undergoing NAC using a single-center cohort analysis. This study not only provides novel evidence supporting the prognostic role of LRG in this specific patient population, but also fills the gaps in previous relevant research, thereby holding substantial potential clinical significance for optimizing the prognostic assessment system for ESCC patients. LRG demonstrated significant prognostic predictive ability independent of other known prognostic variables, showing statistically significant survival advantages in various analyses including univariate and multivariate Cox analyses. This study has significant clinical value, providing effective prognostic biomarkers for ESCC patients, opportunities for different treatment strategies, and the foundation for personalized pathology informatics. Through the risk factor analysis of OS and DFS in ypN(+) patients (**Supplementary Tables 3**–**5**), the degree of nodal regression reflected by LRG still provides additional prognostic stratification value beyond ypN stage in ypN(+) patients with confirmed residual nodal disease.

In many studies, NAC is often considered to improve the prognosis of patients with locally advanced and advanced esophageal carcinoma (18, 19). The prognostic significance of tumor and lymph node downstaging following neoadjuvant treatment in patients with esophageal adenocarcinoma is well established (20). Recent single-center studies investigating the regression of pathological lymph nodes have indicated that esophageal adenocarcinoma patients showing lymph nodes response tend to have better survival outcomes and lower tumor recurrence rates. This association appears to be independent of other prognostic factors, including the degree of primary tumor regression (21, 22). However, previous studies on the role of lymph node regression in prognosis have primarily focused on esophageal adenocarcinoma. This study, however, focuses on the prognostic value of lymph node regression in the diagnosis and treatment of ESCC patients (14).Specifically, distinct from prior research predominantly centered on adenocarcinoma cohorts, the present study provides a systematic evaluation and validation of LRG within an ESCC population. Furthermore, we developed an integrated prognostic nomogram based on LRG combined with clinicopathological features, aiming to facilitate personalized risk stratification and survival prediction for ESCC patients.

A recent study indicated that following NAC treatment, lymph node response (with lymph node shrinkage measured on CT scans being more than 30%) can predict long-term survival in metastatic esophageal adenocarcinoma (23). However, imaging assessments may be challenging due to treatment-induced local inflammation. In addition to imaging evaluations, many studies have shown that persistent positive lymph nodes are a significant adverse prognostic factor after neoadjuvant treatment (24, 25). Furthermore, the number of positive lymph nodes in surgical specimens has been confirmed as an independent adverse prognostic factor for esophageal cancer prognosis (26). However, the full value of lymph node regression in ESCC patients’ prognosis has not been fully elucidated. Nieman et al.’s studies evaluating lymph node pathological responses in esophageal cancer patients after NAC or neoadjuvant chemoradiation therapy have reported that patients with a lymph node reaction and complete remission had a significantly better prognosis than lymph node-negative patients without a reaction (27, 28).These findings align with our analysis results.

Davies et al. studied the relationship between lymph node pathological response post-NAC treatment and survival in esophageal adenocarcinoma, establishing a scoring system based on the ratio of fibrosis and residual tumor within the lymph nodes. Their study showed that patients with a response in the lymph nodes (accounting for 50% of remaining tumor cells) had significantly improved OS and DFS rates, with reduced local and systemic recurrence rates (10). Several studies have evaluated the significant relationship between lymph node regression and prognosis in esophageal cancer (13). The results of the above studies are consistent with the findings of this study.

The survival benefit of lymph node response is independent of the presence of primary tumor regression. Notably, the status of lymph node regression may not always align with that of primary tumor regression, a discrepancy that can be attributed to divergent biological behaviors between primary tumors and metastatic lymph nodes. In this regard, lymph node regression carries prognostic significance distinct from that of primary tumor regression. Multiple studies indicate that pathological lymph node downstaging following neoadjuvant therapy is closely associated with long-term survival and can provide incremental prognostic information beyond ypTNM stage and primary tumor TRG. For instance, Moore et al. confirmed in a multicenter cohort of esophageal adenocarcinoma that lymph node regression was a powerful independent prognostic factor (14). Similarly, a systematic review and meta-analysis by Chen et al. demonstrated that both lymph node regression grade and downstaging of N stage were associated with significantly improved survival (29). From a mechanistic perspective, the immune microenvironment, immune cell infiltration, and drug penetration within tumor-draining lymph nodes differ substantially from those in the primary site. These differences may render lymph nodes a more sensitive indicator of systemic treatment efficacy against metastatic and micrometastatic deposits (30). Against this backdrop, the fact that LRG in our study retained its independent prognostic effect in multivariate analysis, while the significance of some traditional nodal staging parameters was diminished, supports the notion that lymph node regression holds unique informational value in prognosis assessment, distinct from primary tumor response. Factors such as tumor size, drug delivery efficiency, and distinct immune microenvironments may collectively influence the therapeutic efficacy of NAC (21). Our study demonstrated through multivariate Cox analysis that LRG serves as an independent prognostic factor. This finding is consistent with the retrospective study by Takaomi et al,which revealed that compared to primary tumor regression (21), lymph node response exhibited a stronger correlation with prognosis in esophageal cancer patients undergoing NAC followed by surgical resection. With the refinement of NAC responder definitions, novel response grading systems incorporating lymph node regression assessment could enable more precise stratification of patients for personalized therapeutic strategies.

In this study, considering ypTNM, Tumor differentiation, and other pathological features are traditional key prognostic markers for esophageal cancer, we constructed a prognostic nomogram by incorporating Age, Tumor differentiation, ypT stage, ypN stage, and LRG into the model after sensitivity analyses. The model exhibited excellent predictive performance, demonstrating good consistency with the actual survival status of ESCC patients. Similar to our study, a recent study indicated that a staging model combining TRG and lymph node regression outperformed AJCC’s neoadjuvant treatment ypTNM staging for prognosis prediction (31). Other studies have shown that regardless of TRG status, lymph node status after neoadjuvant treatment is the most important prognostic factor for resectable esophageal cancer patients (32). In recent years, several prognostic models for esophageal cancer have been developed using different approaches, including models based on autophagy-related genes, immunological features, or immune genes, and radiomics (33). These different perspectives provide comprehensive information for tumor patients, including the pathological evaluation of lymph node regression status, which may bring more significant prognostic predictive value when combined with multi-omics data in the future to guide prognosis assessment. Given that this study is based on the context of neoadjuvant chemotherapy, extrapolation of our findings to neoadjuvant chemoradiotherapy or neoadjuvant chemoradiotherapy combined with immunotherapy should be made with caution.

This study has several limitations. First, despite our efforts to include all eligible patients, the retrospective, single-center design inherently limits the generalizability of the conclusions. Furthermore, we acknowledge that the three-tier LRG system adopted in this study has relatively limited granularity, and its prognostic discriminatory ability requires further validation in larger samples. In addition, external validation in an independent cohort is currently lacking. Therefore, future large-sample, multicenter prospective studies are needed to further verify the reliability and accuracy of our findings. Second, the overall sample size was relatively limited, and an imbalance in case numbers between groups (e.g., LN-R group vs. control groups) may have affected statistical power and the reliability of subgroup comparisons. Third, for sporadic missing data, we employed mean (for continuous variables) or mode (for categorical variables) imputation. This simple approach may underestimate the true variability of the parameters and constitutes another limitation. Fourth, the prognostic nomogram developed here was only internally validated; external validation with an independent cohort is lacking. Thus, its clinical applicability and generalizability require further confirmation through prospective, multicenter studies with larger samples. Fifth, lymph node specimens were not collected by a single surgeon. Variations in surgical principles among surgeons could influence the quality and extent of lymph node dissection, introducing potential bias. Finally, the assessment of lymph node regression grading (LRG) depends on meticulous pathological review, a process that is somewhat subjective and time-consuming, which may limit its immediate widespread adoption in routine clinical practice.

## Conclusion

In this study, we found that lymph node response and ESCC patients’ prognosis were closely related. The LRG system we develop can serve as an independent prognostic factor for ESCC patients. By combining lymph node response and other clinical characteristics, the precise assessment of patient prognosis can be further improved. These findings provide preliminary evidence for the independent prognostic value of LRG and its nomogram in risk stratification, yet large-scale, multicenter studies are needed for further validation.

## Data Availability

The original contributions presented in the study are included in the article/**Supplementary Material**. Further inquiries can be directed to the corresponding author.
