# Supplementary material for: Refining post-neoadjuvant risk stratification in ESCC with lymph node regression grade
Source: Front Oncol. 2026 May 13;16:1723139. doi: 10.3389/fonc.2026.1723139 (PMC13216772; doi:10.3389/fonc.2026.1723139)
Supplement: Supplementary file 1 [file DataSheet1.docx]

**Supplementary Tables**

**Supplementary Table 1.** Univariate Cox Regression Analysis of the Association Between Clinical Variables and Survival Outcomes

| Variables | Univariate analysis | | |
| --- | --- | --- | --- |
|  | HR | 95%CI | P value |
| **Age** |  |  |  |
| <=60 | 1.00 |  |  |
| >60 | 1.74 | 1.17-2.58 | 0.006 |
| **Sex** |  |  |  |
| Male | 1.00 |  |  |
| Female | 0.99 | 0.60-1.62 | 0.961 |
| **BMI** |  |  |  |
| <=23 | 1.00 |  |  |
| >23 | 1.00 | 0.69-1.49 | 0.956 |
| **Location** |  |  |  |
| Upper | 1.00 |  |  |
| Middle | 0.86 | 0.53-1.38 | 0.528 |
| Low | 0.7 | 0.40-1.28 | 0.260 |
| **ypT stage** |  |  |  |
| 0-2 | 1.00 |  |  |
| 3-4 | 2.19 | 1.49-3.22 | < 0.001 |
| **ypN stage** |  |  |  |
| 0 | 1.00 |  |  |
| 1 | 1.74 | 1.11-2.73 | 0.017 |
| 2 | 4.71 | 2.76-8.05 | < 0.001 |
| 3 | 4.13 | 2.23-7.63 | < 0.001 |
| **Tumor differentiation** | |  |  |
| 1 | 1.00 |  |  |
| 2 | 2.75 | 1.72-4.42 | < 0.001 |
| 3 | 4.18 | 2.63-6.65 | < 0.001 |
| **TRG** |  |  |  |
| 0 | 1.00 |  |  |
| 1 | 1.66 | 0.75-3.66 | 0.209 |
| 2 | 2.49 | 1.24-4.97 | < 0.001 |
| 3 | 4.82 | 2.41-9.64 | < 0.001 |
| **LRG** |  |  |  |
| LN-NR | 1.00 |  |  |
| LN-R | 0.25 | 0.14-0.43 | < 0.001 |
| LN-Neg | 0.3 | 0.20-0.47 | < 0.001 |

HR: Hazard ratio; CI: Confidence Interval; TRG: tumor regression grade; LRG: Lymph node regression grade; LN-NR: Lymph node non-response; LN-R: Lymph node response; LN-Neg: Lymph node negative.

**Supplementary Table 2.** Multivariate Cox Proportional Hazards Regression Analyses for Three Distinct Models

| Variables | Adjust1 | | | | Adjust2 | | | Adjust3 | | |
| --- | --- | --- | --- | --- | --- | --- | --- | --- | --- | --- |
|  | HR | | 95%CI | P value | HR | 95%CI | P value | HR | 95%CI | P value |
| **Age** |  | |  |  |  |  |  |  |  |  |
| <=60 | 1.00 | |  |  | 1.00 |  |  | 1.00 |  |  |
| >60 | 1.22 | | 0.76-1.96 | 0.407 | 1.24 | 0.78-1.97 | 0.359 | 1.28 | 0.80-2.05 | 0.308 |
| **Sex** |  | |  |  |  |  |  |  |  |  |
| Male | 1.00 | |  |  | 1.00 |  |  | 1.00 |  |  |
| Female | 1.16 | | 0.65-2.10 | 0.615 | 1.06 | 0.61-1.84 | 0.829 | 1.23 | 0.69-2.20 | 0.485 |
| **BMI** |  | |  |  |  |  |  |  |  |  |
| <=23 | 1.00 | |  |  | 1.00 |  |  | 1.00 |  |  |
| >23 | 0.87 | | 0.58-1.31 | 0.498 | 0.95 | 0.64-1.41 | 0.800 | 0.82 | 0.55-1.23 | 0.341 |
| **Location** |  | |  |  |  |  |  |  |  |  |
| Upper | 1.00 | |  |  | 1.00 |  |  | 1.00 |  |  |
| Middle | 1.25 | | 0.73-2.14 | 0.422 | 1.23 | 0.73-2.10 | 0.436 | 1.25 | 0.73-2.16 | 0.416 |
| Low | 0.97 | | 0.51-1.87 | 0.930 | 0.96 | 0.52-1.78 | 0.894 | 0.96 | 0.50-1.85 | 0.900 |
| **ypT stage** |  | |  |  |  |  |  |  |  |  |
| 0-2 | 1.00 | |  |  | 1.00 |  |  |  |  |  |
| 3-4 | 1.43 | | 0.86-2.37 | 0.167 | 1.77 | 1.17-2.67 | 0.007 |  |  |  |
| **ypN stage** |  | |  |  |  |  |  |  |  |  |
| 0 | 1.00 | |  |  | 1.00 |  |  | 1.00 |  |  |
| 1 | 1.28 | | 0.64-2.54 | 0.485 | 1.34 | 0.68-2.64 | 0.399 | 1.25 | 0.63-2.46 | 0.518 |
| 2 | 1.84 | | 0.81-4.17 | 0.145 | 1.88 | 0.83-4.25 | 0.131 | 1.93 | 0.86-4.34 | 0.112 |
| 3 | 2.88 | | 1.28-6.52 | 0.011 | 2.66 | 1.18-5.97 | 0.018 | 2.81 | 1.26-6.29 | 0.012 |
| **Tumor differentiation** | |  | |  |  |  |  | |  |  |
| 1 | 1.00 | |  |  | 1.00 |  |  | 1.00 |  |  |
| 2 | 2.16 | | 1.30-3.59 | 0.003 | 2.32 | 1.40-3.84 | 0.001 | 2.08 | 1.25-3.45 | 0.005 |
| 3 | 2.83 | | 1.69-4.75 | < 0.001 | 3.11 | 1.88-5.12 | < 0.001 | 2.69 | 1.61-4.50 | < 0.001 |
| **TRG** |  | |  |  |  |  |  |  |  |  |
| 0 | 1.000 | |  |  |  |  |  | 1.00 |  |  |
| 1 | 0.97 | | 0.41-2.34 | 0.954 |  |  |  | 1.10 | 0.47-2.60 | 0.823 |
| 2 | 1.23 | | 0.54-2.80 | 0.620 |  |  |  | 1.58 | 0.76-3.28 | 0.226 |
| 3 | 1.73 | | 0.71-4.22 | 0.226 |  |  |  | 2.42 | 1.13-5.16 | 0.023 |
| **LRG** |  | |  |  |  |  |  |  |  |  |
| LN-NR | 1.000 | |  |  | 1.00 |  |  | 1.00 |  |  |
| LN-R | 0.41 | | 0.23-0.76 | 0.004 | 0.38 | 0.21-0.68 | 0.001 | 0.44 | 0.24-0.80 | 0.007 |
| LN-Neg | 0.50 | | 0.25-0.99 | 0.048 | 0.49 | 0.24-0.97 | 0.041 | 0.51 | 0.26-1.01 | 0.055 |

HR: Hazard ratio; CI: Confidence Interval; TRG: tumor regression grade; LRG: Lymph node regression grade; LN-NR: Lymph node non-response; LN-R: Lymph node response; LN-Neg: Lymph node negative. Adjust1: all covariates included. Adjust 2: TRG excluded. Adjust 3: ypT stage excluded.

**Supplementary Figure 1.** Correlation Heatmap of Clinicopathological Variables in ESCC Patients


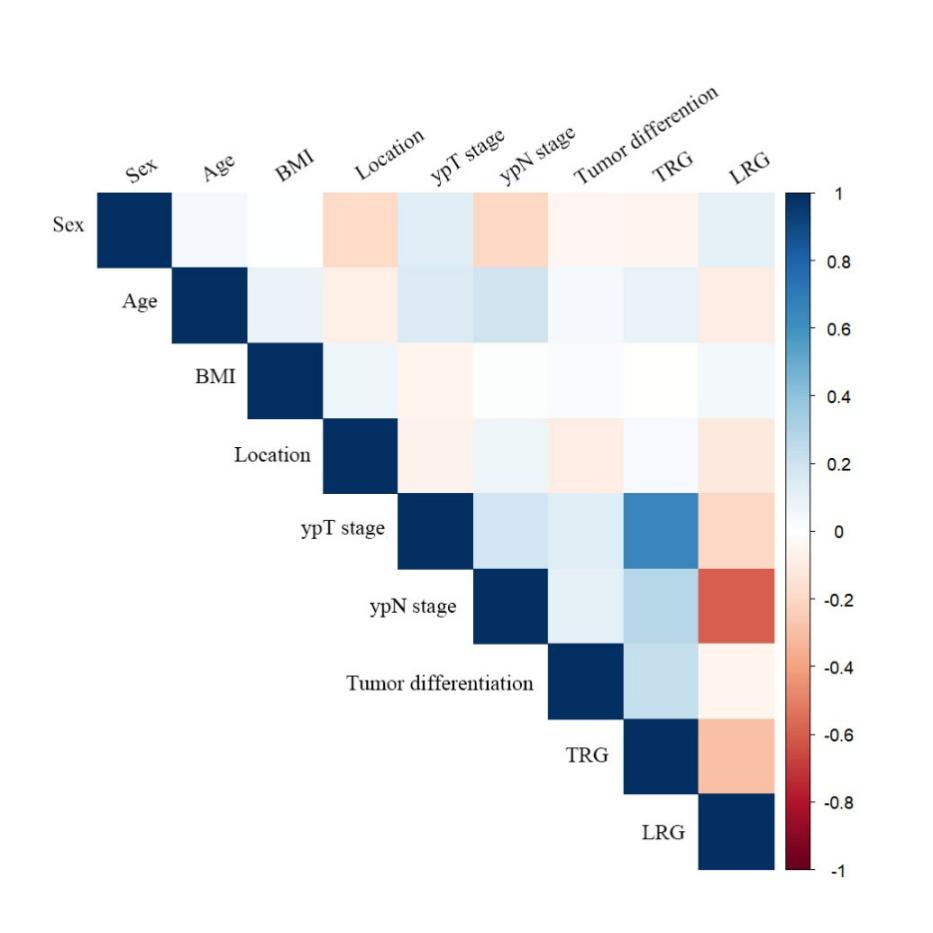


**Supplementary Table 3.** Univariate Survival Analysis of OS and DFS in ypN (+) Patients

| Variables | Univariate analysis(OS) | | | Univariate analysis(DFS) | | |
| --- | --- | --- | --- | --- | --- | --- |
|  | HR | 95%CI | P value | HR | 95%CI | P value |
| **Age** |  |  |  |  |  |  |
| <=60 | 1.00 |  |  | 1.00 |  |  |
| >60 | 1.57 | 0.98-2.52 | 0.059 | 1.45 | 0.84-2.50 | 0.187 |
| **Sex** |  |  |  |  |  |  |
| Male | 1.00 |  |  | 1.00 |  |  |
| Female | 0.96 | 0.49-1.87 | 0.908 | 0.57 | 0.23-1.44 | 0.236 |
| **BMI** |  |  |  |  |  |  |
| <=23 | 1.00 |  |  | 1.00 |  |  |
| >23 | 0.89 | 0.55-1.43 | 0.616 | 0.78 | 0.44-1.37 | 0.384 |
| **Location** |  |  |  |  |  |  |
| Upper | 1.00 |  |  | 1.00 |  |  |
| Middle | 0.97 | 0.53-1.77 | 0.923 | 1.33 | 0.59-3.01 | 0.498 |
| Low | 0.68 | 0.34-1.37 | 0.280 | 1.32 | 0.55-3.18 | 0.540 |
| **ypT stage** |  |  |  |  |  |  |
| 0-2 | 1.00 |  |  | 1.00 |  |  |
| 3-4 | 2.59 | 1.58-4.23 | < 0.001 | 2.44 | 1.37-4.33 | 0.002 |
| **ypN stage** |  |  |  |  |  |  |
| 0 | 1.00 |  |  | 1.00 |  |  |
| 1 | 1.61 | 0.82-3.16 | 0.171 | 1.24 | 0.59-2.62 | 0.575 |
| 2 | 3.71 | 1.75-7.89 | < 0.001 | 2.71 | 1.17-6.27 | 0.020 |
| 3 | 3.26 | 1.43-7.43 | 0.005 | 3.14 | 1.33-7.45 | 0.009 |
| **Tumor differentiation** | |  |  |  |  |  |
| 1 | 1.00 |  |  | 1.00 |  |  |
| 2 | 2.02 | 1.15-3.55 | 0.015 | 2.11 | 1.09-4.09 | 0.028 |
| 3 | 4.32 | 2.51-7.43 | < 0.001 | 4.16 | 2.17-7.94 | < 0.001 |
| **TRG** |  |  |  |  |  |  |
| 0 | 1.00 |  |  | 1.00 |  |  |
| 1 | 2.04 | 0.57-7.33 | 0.276 | 2.16 | 0.45-10.43 | 0.336 |
| 2 | 3.17 | 0.97-10.40 | 0.056 | 3.62 | 0.85-15.35 | 0.081 |
| 3 | 6.32 | 1.92-20.86 | 0.002 | 6.29 | 1.47-26.93 | 0.013 |
| **LRG** |  |  |  |  |  |  |
| LN-NR | 1.00 |  |  | 1.00 |  |  |
| LN-R | 0.23 | 0.13-0.40 | < 0.001 | 0.25 | 0.13-0.50 | < 0.001 |

DFS: Disease-free survival; HR: Hazard ratio; CI: Confidence Interval; TRG: tumor regression grade; LRG: Lymph node regression grade; LN-NR: Lymph node non-response; LN-R: Lymph node response.

**Supplementary Table 4.** Multivariate Cox Regression Analysis of OS in ypN (+) Patients Based on Three Distinct Models

| Variables | Adjust1 | | | | Adjust2 | | | Adjust3 | | |
| --- | --- | --- | --- | --- | --- | --- | --- | --- | --- | --- |
|  | HR | | 95%CI | P value | HR | 95%CI | P value | HR | 95%CI | P value |
| **ypT stage** |  | |  |  |  |  |  |  |  |  |
| 0-2 | 1.00 | |  |  | 1.00 |  |  |  |  |  |
| 3-4 | 2.37 | | 1.33-4.22 | 0.004 | 2.61 | 1.54-4.42 |  |  |  |  |
| **ypN stage** |  | |  |  |  |  |  |  |  |  |
| 0 | 1.00 | |  |  | 1.00 |  |  | 1.00 |  |  |
| 1 | 0.93 | | 0.45-1.89 | 0.833 | 0.97 | 0.48-1.96 | 0.927 | 0.97 | 0.48-2.00 | 0.944 |
| 2 | 1.38 | | 0.62-3.11 | 0.432 | 1.46 | 0.66-3.22 | 0.354 | 1.73 | 0.78-3.86 | 0.181 |
| 3 | 1.91 | | 0.78-4.66 | 0.157 | 1.74 | 0.73-4.15 | 0.211 | 2.02 | 0.82-4.99 | 0.128 |
| **Tumor differentiation** | |  | |  |  |  |  | |  |  |
| 1 | 1.00 | |  |  | 1.00 |  |  | 1.00 |  |  |
| 2 | 1.36 | | 0.74-2.48 | 0.321 | 1.41 | 0.77-4.15 | 0.263 | 1.29 | 0.71-2.37 | 0.404 |
| 3 | 2.63 | | 1.42-4.86 | 0.002 | 2.79 | 1.52-5.10 | < 0.001 | 2.16 | 1.17-3.97 | 0.014 |
| **TRG** |  | |  |  |  |  |  |  |  |  |
| 0 | 1.00 | |  |  |  |  |  | 1.00 |  |  |
| 1 | 0.80 | | 0.21-3.10 | 0.752 |  |  |  | 1.14 | 0.30-4.29 | 0.849 |
| 2 | 1.14 | | 0.31-4.15 | 0.842 |  |  |  | 2.01 | 0.60-7.74 | 0.260 |
| 3 | 1.32 | | 0.35-4.99 | 0.686 |  |  |  | 2.65 | 0.76-9.18 | 0.125 |
| **LRG** |  | |  |  |  |  |  |  |  |  |
| LN-NR | 1.00 | |  |  | 1.00 |  |  | 1.00 |  |  |
| LN-R | 0.34 | | 0.18-0.64 | < 0.001 | 0.33 | 0.18-0.60 | < 0.001 | 0.37 | 0.20-0.71 | 0.003 |

OS: overall survival; HR: Hazard ratio; CI: Confidence Interval; TRG: tumor regression grade; LRG: Lymph node regression grade; LN-NR: Lymph node non-response; LN-R: Lymph node response. Adjust1: all covariates included. Adjust 2: TRG excluded. Adjust 3: ypT stage excluded.

**Supplementary Table 5.** Multivariate Cox Regression Analysis of DFS in ypN (+) Patients Based on Three Distinct Models

| Variables | Adjust1 | | | | Adjust2 | | | Adjust3 | | |
| --- | --- | --- | --- | --- | --- | --- | --- | --- | --- | --- |
|  | HR | | 95%CI | P value | HR | 95%CI | P value | HR | 95%CI | P value |
| **ypT stage** |  | |  |  |  |  |  |  |  |  |
| 0-2 | 1.00 | |  |  | 1.00 |  |  |  |  |  |
| 3-4 | 2.02 | | 1.04-3.92 | 0.038 | 2.02 | 1.04-3.92 | 0.038 |  |  |  |
| **ypN stage** |  | |  |  |  |  |  |  |  |  |
| 0 | 1.00 | |  |  | 1.00 |  |  | 1.00 |  |  |
| 1 | 0.83 | | 0.38-1.81 | 0.638 | 0.83 | 0.38-1.81 | 0.638 | 0.85 | 0.39-1.86 | 0.682 |
| 2 | 1.03 | | 0.42-2.56 | 0.945 | 1.03 | 0.42-2.56 | 0.945 | 1.19 | 0.48-2.94 | 0.702 |
| 3 | 1.74 | | 0.67-4.50 | 0.256 | 1.74 | 0.67-4.50 | 0.256 | 1.80 | 0.68-4.75 | 0.235 |
| **Tumor differentiation** | |  | |  |  |  |  | |  |  |
| 1 | 1.00 | |  |  | 1.00 |  |  | 1.00 |  |  |
| 2 | 1.47 | | 0.72-3.01 | 0.286 | 1.47 | 0.72-3.01 | 0.286 | 1.41 | 0.69-2.85 | 0.346 |
| 3 | 2.48 | | 1.22-5.05 | 0.012 | 2.48 | 1.22-5.05 | 0.012 | 2.29 | 1.13-4.65 | 0.022 |
| **TRG** |  | |  |  |  |  |  |  |  |  |
| 0 | 1.00 | |  |  |  |  |  | 1.00 |  |  |
| 1 | 0.98 | | 0.19-5.05 | 0.983 | 0.98 | 0.19-5.05 | 0.983 | 1.23 | 0.24-6.23 | 0.802 |
| 2 | 1.43 | | 0.30-6.72 | 0.654 | 1.43 | 0.30-6.72 | 0.654 | 2.13 | 0.48-9.43 | 0.319 |
| 3 | 1.58 | | 0.31-7.97 | 0.579 | 1.58 | 0.31-7.97 | 0.579 | 2.87 | 0.64-13.00 | 0.170 |
| **LRG** |  | |  |  |  |  |  |  |  |  |
| LN-NR | 1.00 | |  |  | 1.00 |  |  | 1.00 |  |  |
| LN-R | 0.38 | | 0.18-0.82 | 0.013 | 0.38 | 0.18-0.82 | 0.013 | 0.42 | 0.19-0.91 | 0.029 |

DFS: Disease-free surviva; HR: Hazard ratio; CI: Confidence Interval; TRG: tumor regression grade; LRG: Lymph node regression grade; LN-NR: Lymph node non-response; LN-R: Lymph node response. Adjust1: all covariates included. Adjust 2: TRG excluded. Adjust 3: ypT stage excluded.

**Supplementary Table 6.** Multivariate Cox Regression Analysis of OS Incorporating the Total Number of Lymph Nodes Dissected

| Variables | Multivariate analysis | | |
| --- | --- | --- | --- |
|  | HR | 95%CI | P value |
| **Age** |  |  |  |
| <=60 | 1.00 |  |  |
| >60 | 1.26 | 0.80-1.99 | 0.320 |
| **Total number of lymph nodes dissected** | | | |
|  | 1.00 | 0.98-1.01 | 0.757 |
| **ypT stage** |  |  |  |
| 0-2 | 1.00 |  |  |
| 3-4 | 1.53 | 0.93-2.50 | 0.092 |
| **ypN stage** |  |  |  |
| 0 | 1.00 |  |  |
| 1 | 1.31 | 0.66-2.58 | 0.436 |
| 2 | 1.84 | 0.83-4.09 | 0.134 |
| 3 | 2.77 | 1.23-6.23 | 0.014 |
| **Tumor differentiation** | |  |  |
| 1 | 1.00 |  |  |
| 2 | 2.11 | 1.28-3.50 | 0.004 |
| 3 | 2.72 | 1.60-4.60 | < 0.001 |
| **TRG** |  |  |  |
| 0 | 1.00 |  |  |
| 1 | 0.94 | 0.40-2.23 | 0.890 |
| 2 | 1.14 | 0.51-2.57 | 0.748 |
| 3 | 1.55 | 0.66-3.65 | 0.317 |
| **LRG** |  |  |  |
| LN-NR | 1.00 |  |  |
| LN-R | 0.40 | 0.22-0.74 | 0.004 |
| LN-Neg | 0.52 | 0.27-1.03 | 0.062 |

OS: overall survival; HR: Hazard ratio; CI: Confidence Interval; TRG: tumor regression grade; LRG: Lymph node regression grade; LN-NR: Lymph node non-response; LN-R: Lymph node response; LN-Neg: Lymph node negative.

**Supplementary Table 7.** Multivariate Cox Regression Analysis of DFS Incorporating the Total Number of Lymph Nodes Dissected

| Variables | Multivariate analysis | | |
| --- | --- | --- | --- |
|  | HR | 95%CI | P value |
| **Age** |  |  |  |
| <=60 | 1.00 |  |  |
| >60 | 1.29 | 0.72-2.29 | 0.387 |
| **Sex** |  |  |  |
| Male | 1.00 |  |  |
| Female | 0.81 | 0.35-1.85 | 0.615 |
| **BMI** |  |  |  |
| <=23 | 1.00 |  |  |
| >23 | 0.76 | 0.45-1.29 | 0.305 |
| **Location** |  |  |  |
| Upper | 1.00 |  |  |
| Middle | 1.74 | 0.80-3.77 | 0.164 |
| Low | 2.04 | 0.86-4.83 | 0.1.4 |
| **Total number of lymph nodes dissected** | | | |
|  | 0.99 | 0.97-1.01 | 0.263 |
| **ypT stage** |  |  |  |
| 0-2 | 1.00 |  |  |
| 3-4 | 1.73 | 0.93-3.21 | 0.081 |
| **ypN stage** |  |  |  |
| 0 | 1.00 |  |  |
| 1 | 1.12 | 0.52-2.44 | 0.775 |
| 2 | 1.10 | 0.43-2.84 | 0.838 |
| 3 | 2.17 | 0.86-5.48 | 0.100 |
| **Tumor differentiation** | |  |  |
| 1 | 1.00 |  |  |
| 2 | 1.58 | 0.83-2.98 | 0.161 |
| 3 | 2.03 | 1.05-3.94 | 0.036 |
| **TRG** |  |  |  |
| 0 | 1.00 |  |  |
| 1 | 1.87 | 0.54-6.47 | 0.322 |
| 2 | 1.37 | 0.42-4.47 | 0.603 |
| 3 | 1.88 | 0.54-6.60 | 0.321 |
| **LRG** |  |  |  |
| LN-NR | 1.00 |  |  |
| LN-R | 0.23 | 0.10-0.53 | < 0.001 |
| LN-Neg | 0.99 | 0.97-1.01 | 0.263 |

DFS: Disease-free surviva;HR: Hazard ratio; CI: Confidence Interval; TRG: tumor regression grade; LRG: Lymph node regression grade; LN-NR: Lymph node non-response; LN-R: Lymph node response; LN-Neg: Lymph node negative.

**Supplementary Table 8.**Summary of Events in the Total Cohort: Statistics of OS and DFS Endpoint Events

| **Endpoint** | **Total Cases** | **Number of Events** | **Event Rate** | **Median Follow-Up Time** |
| --- | --- | --- | --- | --- |
| OS | 173 | 112 | 64.7% | 74 momths |
| DFS | 173 | 70 | 40.5% | 62 momths |

OS: overall survival;DFS: Disease-free surviva.

**Supplementary Figure 2.** Decision curve analysis for 3-year OS prediction


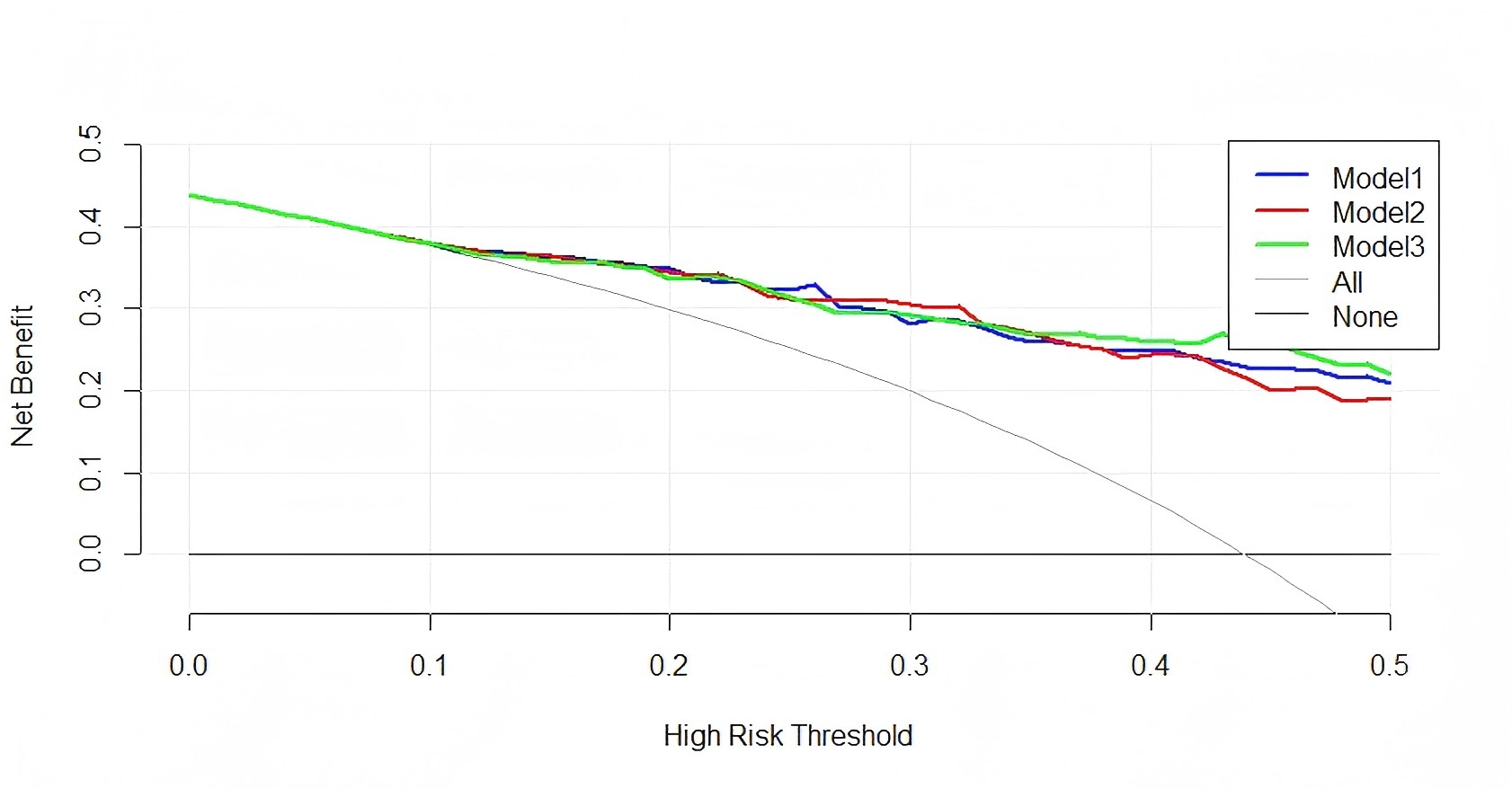


OS: overall survival.Model 1: all covariates included. Model 2: TRG excluded. Model 3: ypT stage excluded.

**Supplementary Figure 3.** Decision curve analysis for 5-year OS prediction


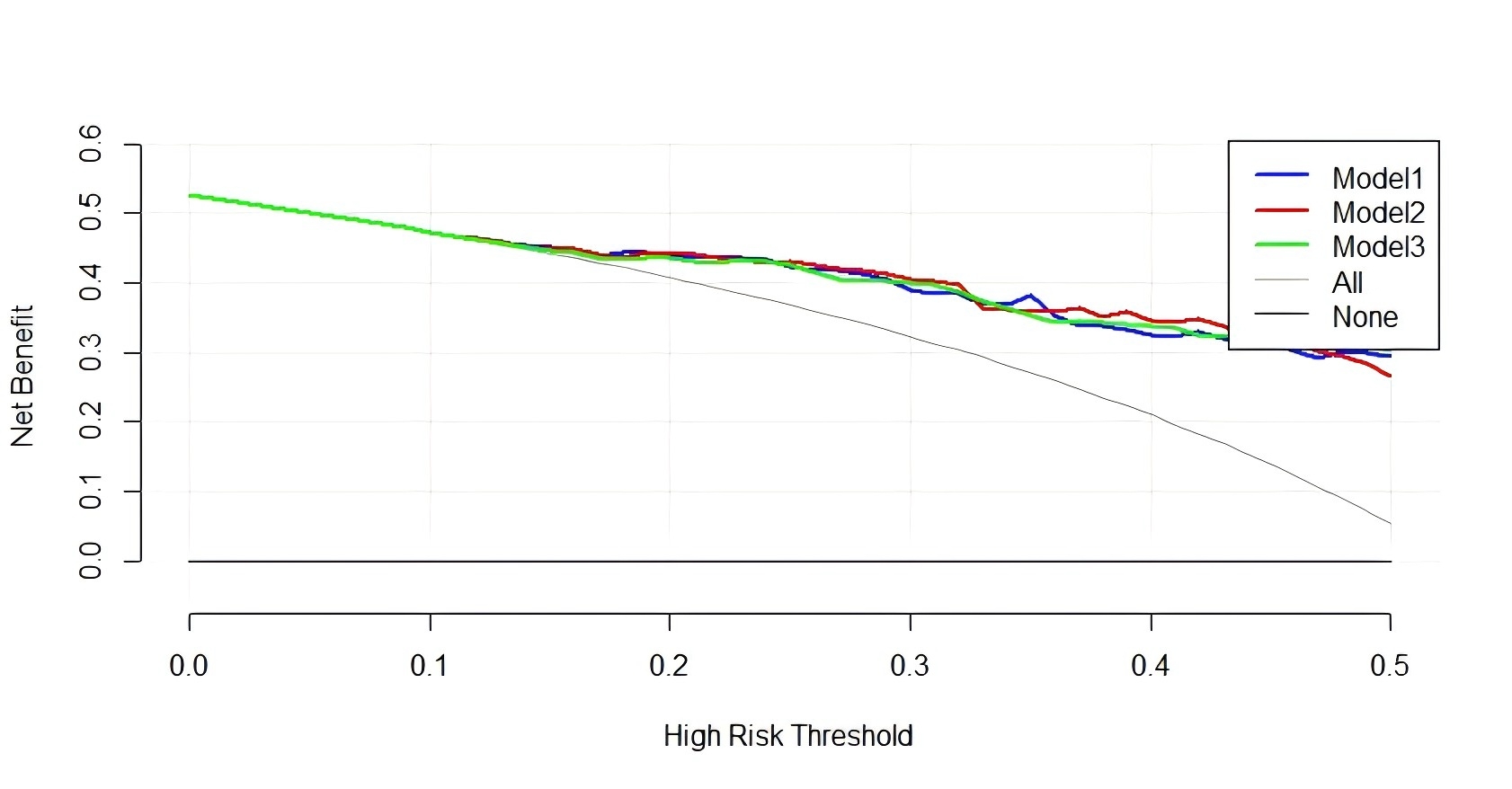


OS: overall survival.aModel 1: all covariates included. Model 2: TRG excluded. Model 3: ypT stage excluded.
